# Glucocorticoids and Antivirals for HBV Reactivation in Onco-Hematologic Patients.

**DOI:** 10.4084/MJHID.2010.035

**Published:** 2010-12-13

**Authors:** Alfredo Marzano, Andrea Marengo, Michela di Fonzo, Paola Begini, Antonella Ferrari, Bruno Monarca, Gianfranco delle Fave, Massimo Marignani

**Affiliations:** 1 Gastroenterology and Hepatology, San Giovanni Battista Hospital, Turin, Italy. Corso Bramante 88, 10125 Torino, Italy; 2 Digestive and Liver Disease Dpt., Second Medical Faculty, University of Rome “La Sapienza” at S. Andrea Hospital. Via di Grottarossa 1035-1039, 00189 Rome, Italy; 3 Hematology Dpt., Second Medical Faculty University of Rome “La Sapienza” at S. Andrea Hospital. Via di Grottarossa 1035-1039, 00189 Rome, Italy

## Abstract

Patients with inactive or occult hepatitis B virus infection and onco-hematological malignancies are at risk of hepatitis flare, hepatic failure and death due to chemotherapy-mediated reactivation. Nucleot(s)ide analogues can reduce reactivation risks and/or hepatitis. However, immuno-mediated phenomena combine to determine liver damage and clinical outcome. We describe in this report two patients with onco-hematological malignancies and hepatitis B reactivation after chemotherapy in whom glucocorticoids were added to nucleot(s)ide. Antiviral therapy was effective on replication, while glucocorticoids managed hyperergic response. One patient without underlying liver disease survived, while the second died and the autopsy demonstrated cirrhosis undetected before death. This clinical trial suggests that in patients with onco-hematological malignancies and altered liver function tests in spite of effective antiviral response, glucocorticoids could control the effects of immune response. However prognosis and survival are related to the underlying liver status.

## Introduction:

In overt or occult HBV carriers with onco-hematological malignancies, viral reactivation can be a consequence of chemotherapy, influencing the risk of developing hepatitis flare, hepatic failure and death.^1^ Nucleot(s)ide analogues (NUC) are used to reduce the risk of reactivation in overt carriers controlling viral replication. Prophylaxis in HBsAg-negative/anti-HBc-positive remains controversial.^1^

Nevertheless, immuno-mediated phenomena, occurring as the result of the host’s immune system reconstitution after chemotherapy, also concur to determine the extent of liver damage and clinical outcome.^2^ No clear indications exist for the management of patients with HBV reactivation in whom biochemical disorders remain and the clinical course deteriorates, in spite of an effective control over viral replication obtained with NUC, possibly as the result of this immuno-mediated hyperergic response.^2^ Glucocorticoids have been sporadically used to control the immuno-mediated effects of HBV reactivation,^3^,^4^ but their role in the management strategies of patients with onco-hematological malignancies has not been defined. Underlying liver disease status could also influence clinical events and survival, with more advanced liver disease determining the worst outcomes.^1^ Our report consists of two patients with onco-hematological malignancies and HBV reactivation in whom glucocorticoids were added to NUC to manage reactivation.

## Case reports

### Case 1:

In July 2006 a 73 year-old man was diagnosed with myelo-monoblastic acute leukemia (AML-M4). Before chemotherapy, ultrasound (US) study of the liver, serum alanine aminotransferase (ALT), total bilirubin, International Normalized Ratio (INR) were normal; anti-HCV, HBsAg and HBV-DNA were negative, anti-HBc, anti-HBs and anti-HBe positive. HBV monitoring was instituted [1]. FLANG (fludarabine, aracytin, novantrone) was administered (August 2006–January 2007), which resulted in the patient going into remission. The patient did not attend the scheduled follow up visit for personal reasons, and in July 2007, sero-reversion to HBsAg and increased ALT levels were detected and he was admitted to the Liver Unit. Table 1 summarizes disease course. ALT were >15 times higher than the upper normal value, HBV-DNA positive (266,700 IU/mL), US study of the liver was normal. High dose lamivudine (LAM, 300 mg/day) was started immediately due to significant viremia and the diagnosis of leukemia. Twenty days later bilirubin, ALT and INR increased in spite of HBV-DNA decrease to 31,000 IU/mL; firstly Adefovir (ADV) and then prednisolone (PDN, 50 mg/day) were added to LAM, speculating that a hyperergic immune response was the cause of liver damage and jaundice. ADV (10 mg/day) was introduced to control mutants eventually escaping LAM. ALT, bilirubin, and INR began to improve after three days with the introduction of PDN. HBV-DNA became negative one month later, and biochemical profile normalized after two months of treatment. During this period LAM and PDN were reduced to 100 and 30 mg/day respectively, and ADV continued. Thereafter, PDN was further reduced and discontinued in November, maintaining LAM and ADV. In May 2008, the patient reverted to HBsAg-negative and anti-HBs positive (9,5 UI/mL). NUC were discontinued on September 2009. One year later the patient is in hematological remission, while both HBsAg and HBV-DNA remain negative.

### Case 2:

In September 2006 a 73 year-old man was diagnosed with follicular lymphoma (grade-3a, stage III). Past medical history was unremarkable and baseline US liver study was normal, ALT were normal, HBsAg and anti-HBe positive, and HBV-DNA persistently below 2,000 IU/mL. He was categorized as inactive carrier and started on LAM before chemotherapy (6R-MINE: rituximab, mesna, ifosfamide, mitroxantrone, etoposide), taken until March 2007. During LAM therapy hematological remission was obtained, ALT were normal, while bilirubin and HBV-DNA remained slightly elevated. In July 2007, the patient continued the LAM treatment, and was admitted to the Liver Unit due to the development of jaundice. Lymphoma relapse was ruled out, but signs of viral and biochemical breakthroughs were present. Table 2 summarizes the disease course. Hepatic failure, mild portal encephalopathy, and ascites developed. LAM resistance (L80V, L180M, M204I) was demonstrated by genotypic analysis (INNO-LiPA DR2), and ADV (10 mg/day) was added. Two weeks later viremia significantly decreased but bilirubin, INR and ALT increased. The addition of PDN (50 mg/day) determined a slight ALT decrease, however bilirubin and INR progressively worsened and the patient died because of irreversible liver failure. The autopsy revealed liver cirrhosis.

## Discussion:

During chemotherapy administration for onco-hematological malignancies, HBV replication can reactivate in overt or occult HBV carriers. Active viral replication reappears during immunosuppression, and hepatocytes infection occurs.^5^ After the restoration of immune response, immune-mediated destruction of infected hepatocytes can cause hepatitis, hepatic failure and even death.^2^ Pre-emptive LAM treatment can reduce reactivation in HBsAg-positive, while universal prophylaxis in HBsAg-negative/anti-HBc-positive remains controversial.^1^ The available data regarding HBV reactivation rates in these patients range from 12% to 25%,^6^–^8^ and have been obtained mainly from studies conducted on B-cell lymphoma patients, many of whom had been treated with the anti CD20 monoclonal antibody rituximab. In this setting LAM usually controls HBV replication and disease evolution.^1^,^6^,^7^ However, a severe course despite adequate inhibition of viral replication can occur.^7^,^9^–^10^ In fact, in the study by Yeo, the reported mortality in lymphoma patients sero-reverting to HBsAg was 20%, despite LAM treatment.^7^ In this study HBV reactivation was diagnosed when an increase in ALT (3-fold or greater or more than 100 IU/L) was associated to HBsAg seroreversion and HBV-DNA increase.^7^ Surveillance protocols based on HBV-DNA and anti-HBs level monitoring have been proposed to detect reactivation of viral replication at an earlier stage.^8^ In the recent Niitsu paper, this approach allowed to detect virological reactivation before ALT increase, even prior to HBsAg reappearance. Patient in whom HBV reactivation was detected by this surveillance protocol were treated with entecavir, a more potent antiviral, and none developed acute hepatitis or died of liver-related causes.^8^ Nevertheless, the cost-effectiveness of this approach, based on high cost technologies and medications, remains to be determined. At any rate, delayed detection of HBV reactivation, and the consequent unnoticed progression to acute hepatitis should be considered a risk factor for a worse clinical outcome, due to the late commencement of treatment,^7^ as it happened in patient 1 due to his inability to attend the follow up appointments. Similarly, the late identification of HBV mutants by genotypic analysis in suboptimal responders to NUC plays a pivotal role, delaying appropriate rescue therapy and supporting HBV reactivation, as it happened in patient 2. Therefore, these situations should be avoided, appropriate surveillance protocols should be developed, enforcing patients to adhere to follow up. Glucocorticoids addition could be useful in this clinical setting^3^–^5^ as these drugs blunt T-cell response targeting HBV-infected hepatocytes.^11^

Glucocorticoids use in the management of HBV reactivation remains anecdotal. Early glucocorticoids administration to manage HBV reactivation has been described in a heterogeneous group of patients, some with onco-hematological malignancies.^3^,^4^ In these trials pre-treatment baseline HBV-DNA and liver disease status were unknown, no case of isolated anti-HBc positive sero-reversion was included, management protocols varied widely, different NUC were used with a variable sequence of glucocorticoids and NUC introduction.^3^,^4^ Nevertheless, these reports seem to suggest that a key strategy is the timely consideration of glucocorticoids addition in those cases in which liver functional status worsens in spite of good virological response to NUC. In this case, adding glucocorticoids might combat the harmful effects of immune reconstitution against HBV infected hepatocytes in onco-hematological malignancies, since NUC alone might not be able to control the progression to liver failure resulting from hyperergic reactions.^9^,^10^ As HBV contains a glucocorticoid-responsive element that may enhance viral replication favoring diffusion and the possible emergence of resistant strains,^12^ these drugs should be introduced when one or more NUC have been started to control replication.

What emerges from our experience, is that the presence of underlying liver disease strongly influences survival, even when a compound treatment (virologic and immunologic) is adopted. Thus, a careful evaluation of HBV markers, clinical history and direct/indirect signs of silent liver disease is mandatory in all onco-hematological malignancies patients eligible for chemotherapy.^1^

## Conclusion:

Our experience supports the policy of baseline screening of onco-hematological malignancies patients for HBV and liver disease status.^1^ Prophylaxis is indicated in inactive carriers, and could be considered in anti-HBc positive subjects with onco-hematological malignancies and/or bone marrow transplantation. When biochemical changes do not improve in spite of effective virologic response to NUC(s), glucocorticoids addition should be considered early in order to control hyperergic immune response. However, prognosis is strictly related to the underlying liver disease status, and to the timely addition of glucocorticoids.

## Figures and Tables

**Table 1.** Timeline of disease evolution. Case 1.

**Date**	**ALT (U/l)**	**Bilirubin (mg/dl)**	**Prothrombin time (INR)**	HBV DNA (IU/ml)*	**Therapy**
17/07/2007	610	1,7	1,1	266.700	LAM 300 mg
20/07/ 2007	767	1,5	1,7	119.700	
25/07/2007	1367	2,5	1,9	45.255	

08/08/2007	2004	12,22	2	31.000	LAM 300 mg + ADV 10 mg + PDN 50 mg
13/08/ 2007	875	13,5	1,4	Nd	
16/08/ 2007	732	8,8	1,07	Nd	
23/08/ 2007	572	5,4	0,9	1.963	

28/08/ 2007	333	3,3	0,9	Nd	LAM 100 mg + ADV 10 mg + PDN 30 mg
19/09/2007	96	1,8	1.1	Negative	
28/10/ 2007	22	1,2	0.9	Negative	

ALT: serum alanine aminotransferase; INR: international normalized ratio; LAM: lamivudine; ADV: adefovir dipivoxil; PND: prednisolone; Nd: Not determined;

* Amplicor Roche PCR, Lower limit of detection <40 IU/ml.

**Table 2.** Timeline of disease evolution. Case 2.

**Date**	**ALT (U/l)**	**Bilirubin (mg/dl)**	**Prothrombin time (INR)**	HBV DNA* (IU/ml)	**Therapy**
9/03/2007	21	2.1	1.14	450	LAM 100 mg
10/07/2007	122	8.0	1.23	Nd	
30/07/2007	200	27.3	1.6	1.470.000	

2/08/2007	190	30.2	1.81	Nd	LAM 100 mg + ADV 10 mg

21/08/2007	169	39.9	2.36	16.024	LAM 100 mg+ ADV 10 mg + PDN 50 mg

23/08/2007	181	42.5	2.26	Nd	LAM 100 mg + ADV 10 mg + PDN 40 mg
26/08/2007	177	48.8	2.14	Nd	
28/08/2007	166	49.7	2.29	Nd	

30/08/2007	136	55.4	2.66	Nd	LAM 100 mg + ADV 10 mg + PDN 30 mg
1/09/2007	132	55.7	3.30	Nd	
2/09/2007	148	56.3	3.50	Nd	**Death**

ALT: serum alanine aminotransferase; INR: international normalized ratio; LAM: lamivudine; ADV: adefovir dipivoxil; PDN: prednisolone; Nd: not determined;

* Amplicor Roche PCR, Lower limit of detection <40 IU/ml.
